# Identification of an Alternating-Access Dynamics Mutant of EmrE with Impaired Transport

**DOI:** 10.1016/j.jmb.2019.05.035

**Published:** 2019-07-12

**Authors:** Chao Wu, Samantha A. Wynne, Nathan E. Thomas, Eva-Maria Uhlemann, Christopher G. Tate, Katherine A. Henzler-Wildman

**Affiliations:** 1 Department of Biochemistry and Molecular Biophysics, Washington University School of Medicine in St. Louis, MO 63110, USA; 2 MRC Laboratory of Molecular Biology, Cambridge CB2 0QH, UK; 3 Department of Biochemistry, University of Wisconsin-Madison, Madison, WI 53706, USA

**Keywords:** Transport, alternating-access, structure, dynamics, NMR, ITC, isothermal titration calorimetry, TPP^+^, tetraphenylphosphonium, MeTPP^+^,  methyltriphenylphosphonium, EtTPP^+^,  ethyltriphenylphosphonium

## Abstract

Proteins that perform active transport must alternate the access of a binding site, first to one side of a membrane and then to the other, resulting in the transport of bound substrates across the membrane. To better understand this process, we sought to identify mutants of the small multidrug resistance transporter EmrE with reduced rates of alternating access. We performed extensive scanning mutagenesis by changing every amino acid residue to Val, Ala, or Gly, and then screening the drug resistance phenotypes of the resulting mutants. We identified EmrE mutants that had impaired transport activity but retained the ability to bind substrate and further tested their alternating access rates using NMR. Ultimately, we were able to identify a single mutation, S64V, which significantly reduced the rate of alternating access but did not impair substrate binding. Six other transport-impaired mutants did not have reduced alternating access rates, highlighting the importance of other aspects of the transport cycle to achieve drug resistance activity *in vivo.* To better understand the transport cycle of EmrE, efforts are now underway to determine a high-resolution structure using the S64V mutant identified here.

## Highlights

• Transporters alternate access of binding sites to move substrates across membranes.
• We sought alternating access-impaired mutants of EmrE through scanning mutagenesis.
• NMR confirms S64V EmrE alternates access ≥ 8-fold slower than WT EmrE.
• Reduced dynamics of S64V EmrE will aid determination of a high-resolution structure.

## Graphical Abstract

**Figure:**
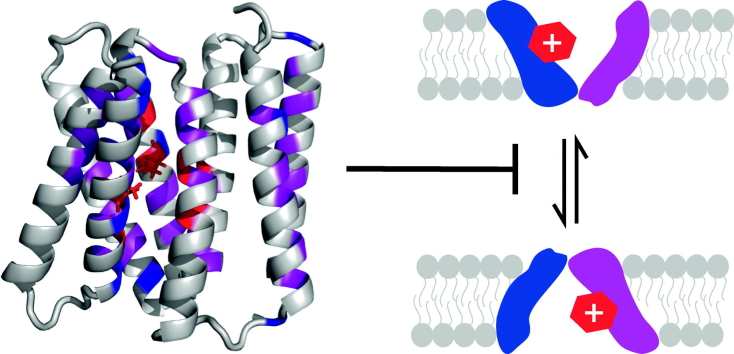

## Introduction

**Fig. 1:**
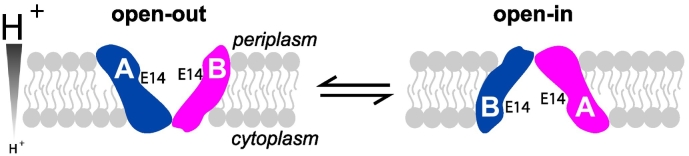
Alternating-access of EmrE. EmrE binds both protons and polyaromatic cation substrates at a shared binding site defined by the two E14 residues from the two protomers of the functional homodimer. Alternating accessibility of this binding site to either side of the membrane occurs when the two protomers in the antiparallel homodimer swap conformational states (illustrated by the different shapes drawn for each protomer). EmrE couples proton import to export of toxic substrates, conferring resistance to these compounds to *E. coli*.

The small multidrug resistance transporter EmrE harnesses the energy of the proton motive force to confer resistance to a wide array of toxic cations [1]*.* EmrE functions as an antiparallel homodimer, with a shared binding site for both protons and drug substrates [2], [3], [4], [5]. The dynamic process of switching conformations so that this binding site alternates between inward facing and outward facing is the key step in moving substrates across the membrane (Fig. 1). When this alternating access process is prevented by specific cross-linking, EmrE is rendered nonfunctional, providing experimental confirmation that alternating access is required for transport [6].

Multiple laboratories have investigated the impact of individual mutations on EmrE function. Several common themes emerged from these studies, such as the importance of Glu14 as a shared binding site both for drugs and protons, the role of transmembrane helices 1, 2, and 3 (TMs 1–3) as the core substrate binding domain, and the role of TM4 as a dimerization domain. These studies identified specific residues critical for drug binding, drug specificity, proton–drug coupling, and dimerization [2], [7], [8], [9], [10], [11], [12], [13], [14], [15], [16], [17]. However, no individual sites have previously been identified as critical for alternating access.

EmrE is the most studied member of the small multidrug resistance family and is the only small multidrug resistance transporter for which even modest resolution structures are available [3], [4], [18]. These structures provided crucial insight into the surprising asymmetric, antiparallel topology of EmrE, but the lack of side chain information hinders a deeper understanding of the transport mechanism. More recently, two models of the EmrE structure have been published [19], [20] based on molecular dynamics simulations of EmrE embedded in lipid bilayers. While these models provide useful information on the transport cycle [19] and dimerization [20] of EmrE, they are ultimately based on the earlier low-resolution experimental structures. There are several potential reasons why a high-resolution structure has proven elusive. First, EmrE is quite small, with short loops connecting four transmembrane helices in only 110 total amino acids. The lack of soluble domains hampers three-dimensional crystallization, and strategies that have proven useful for solving the structure of other integral membrane proteins have been unsuccessful for EmrE. Second, EmrE is inherently dynamic. In fact, there is no known condition under which wild-type EmrE is not dynamic; alternating access occurs even when neither proton nor drug is bound [21], [22], [23], [24]. This natural plasticity is proposed to be an important property enabling its very broad multidrug binding capability [23] but is problematic for the determination of high-resolution structures. Therefore, we have a second motivation in identifying a mutant that suppresses the inherently dynamic nature of EmrE in order to determine a higher resolution structure and better understand how this small protein interacts with such a broad class of substrates.

Here, we identify a critical residue in EmrE, S64V, which alters the rate of alternating access but does not significantly change the affinity for substrate binding.

## Results

### Identification of functionally important residues in EmrE

To efficiently screen for potential dynamics mutants, we took advantage of the fact that alternating access is required for transport. Thus, we sought to identify EmrE point mutations that inhibited transport, as measured by inability to confer resistance to toxic compounds when expressed in *Escherichia coli*. First, wild-type EmrE was mutated at every residue (except the N-terminal Met) to the amino acids Ala, Gly, or Val as described in Materials and Methods. In a few positions, Leu was introduced if the native amino acid was already Ala, Gly, or Val. Previous scanning mutagenesis of EmrE used Cys or Trp substitutions to probe residue accessibility, identify the dimer interface, and insert spin labels for electron paramagnetic resonance studies [8], [9], [11], [13], [16], [25], [26]. However, Ala, Gly, and Val are more common in transmembrane helical regions than Cys or Trp [27], [28]. Substitutions with these three amino acids are well tolerated throughout a membrane protein and have been used previously to isolate conformational specific mutants of the tetracycline carrier TetA(B) [29]. After two rounds of mutagenesis, all but five mutants were made (F44A, W63 V, F79G, D84A, I88G). The EmrE mutants were then tested for their ability to confer resistance to three canonical EmrE substrates: methyl viologen, acriflavine, and ethidium bromide (Figs. S1–S3, Supplementary Data Table 1). Two concentrations of each substrate (see Materials and Methods) were chosen for testing. These concentrations were selected so that *E. coli* strain JM109 containing the plasmid pKK223-3 was unable to grow even at the lower concentration and leaky expression of wild-type *emrE* from the same plasmid was still able to confer resistance and permit growth of JM109 at the higher concentration. Growth was scored as robust at both concentrations of a given substrate (++), growth at both substrate concentrations but notably reduced relative to WT at the higher concentration (+), growth only at the lower substrate concentration (+/−), or no growth at either substrate concentration (−) for each of the three different substrates.

**Fig. 2:**
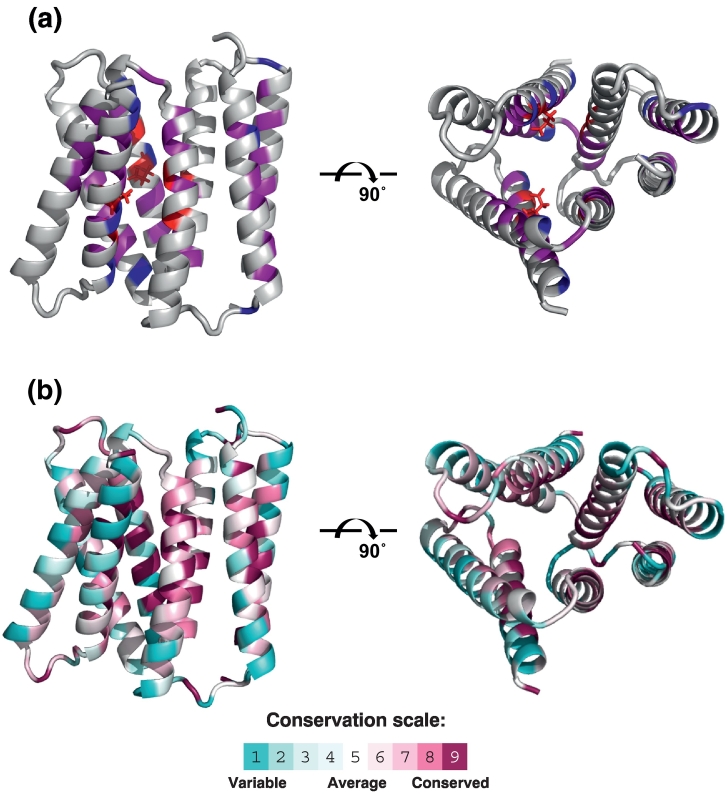
Functionally critical residues in EmrE identified from screening are evolutionarily conserved. (A) Summary of plate assay results from Supplementary Data Table 1 mapped on to the complete structural model of EmrE [19]. Gray, no effect (++) of mutation to V, A, or G at that position on resistance to any of the tested compounds. Blue, modest effect (+) of mutation on resistance profile. Magenta, significant effect (+/−) of mutation on resistance profile. Red, severe defect (−) in growth or resistance to all tested compounds upon mutation of that position to any other amino acid. (B) Evolutionary conservation profile calculated using ConSurf. Functionally critical residues in EmrE identified from screening are evolutionarily conserved. (a) Summary of plate assay results from Supplementary Data Table 1 mapped on to the complete structural model of EmrE [19]. Gray, no effect (++) of mutation to V, A, or G at that position on resistance to any of the tested compounds. Blue, modest effect (+) of mutation on resistance profile. Magenta, significant effect (+/−) of mutation on resistance profile. Red, severe defect (−) in growth or resistance to all tested compounds upon mutation of that position to any other amino acid. (b) Evolutionary conservation profile calculated using ConSurf.

**Fig. 3:**
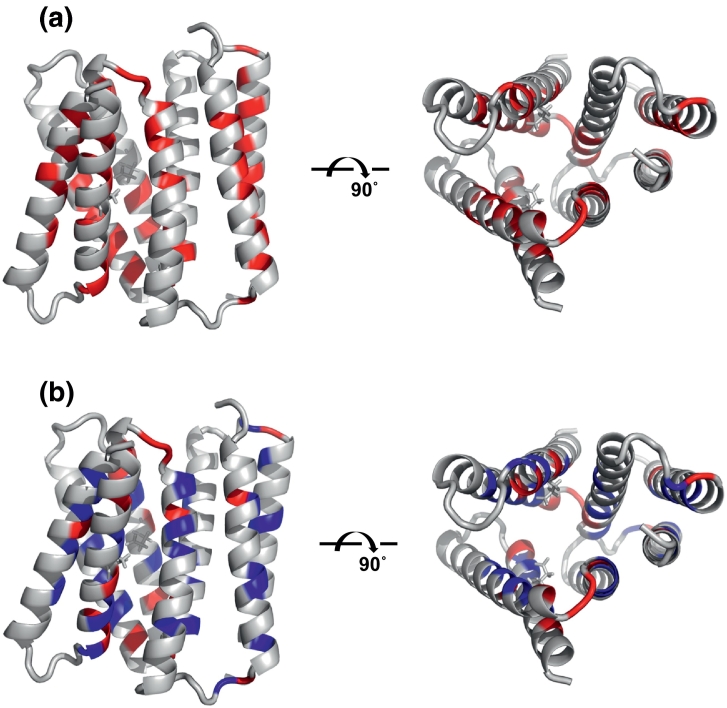
Locations critical for function depend on identity of substituted amino acid and identity of drug. (a) Locations where the identity of the substituted amino acid affects the ethidium bromide resistance phenotype highlighted in red. (b) Locations where different drug substrates led to different growth phenotypes for one of the substituted amino acids (blue) or multiple amino acids (red).

Mapping the transport activity of the EmrE mutants onto the recent all-atom model[19] of EmrE (Fig. 2a) highlights the similarity between the functionally important residues identified experimentally and regions of high-sequence conservation as identified using ConSurf (Fig. 2b). The screen identified residues in TM1–3 lining the substrate binding pocket known to be important for drug and proton binding (e.g., Glu14, Leu47, and Trp63) [2], [11], [12], as well as residues in the TM4 dimerization interface (Gly90, Ile94, and Gly97) [10], [13], [20]. As expected, many locations were sensitive to the identity of the substituted amino acid (Fig. 3a). For example, mutation of Ala10—located just one helical turn away from the Glu14 binding site—to the bulkier valine abolished resistance to all three substrates, while mutation to glycine did not impair the resistance phenotype. In addition, by testing growth on three different EmrE substrates, our scan highlights residues where mutation has a potential effect on drug selectivity (Fig. 3b). Many residues thought to interact with substrates, such as Leu7 and Phe44 [8], conferred varying levels of resistance to different drugs. This supports the hypothesis that substrates with widely variant structures do not necessarily interact with identical amino acid residues and could affect the EmrE structural intermediates differently [30]. Interestingly, a number of residues from the TM2–3 loop and from TM4 had an effect on substrate selectivity, even though these regions are not part of the previously identified substrate binding site [2], [3], [4], [5].

### Identification of potential EmrE dynamics mutants

**Fig. 4:**
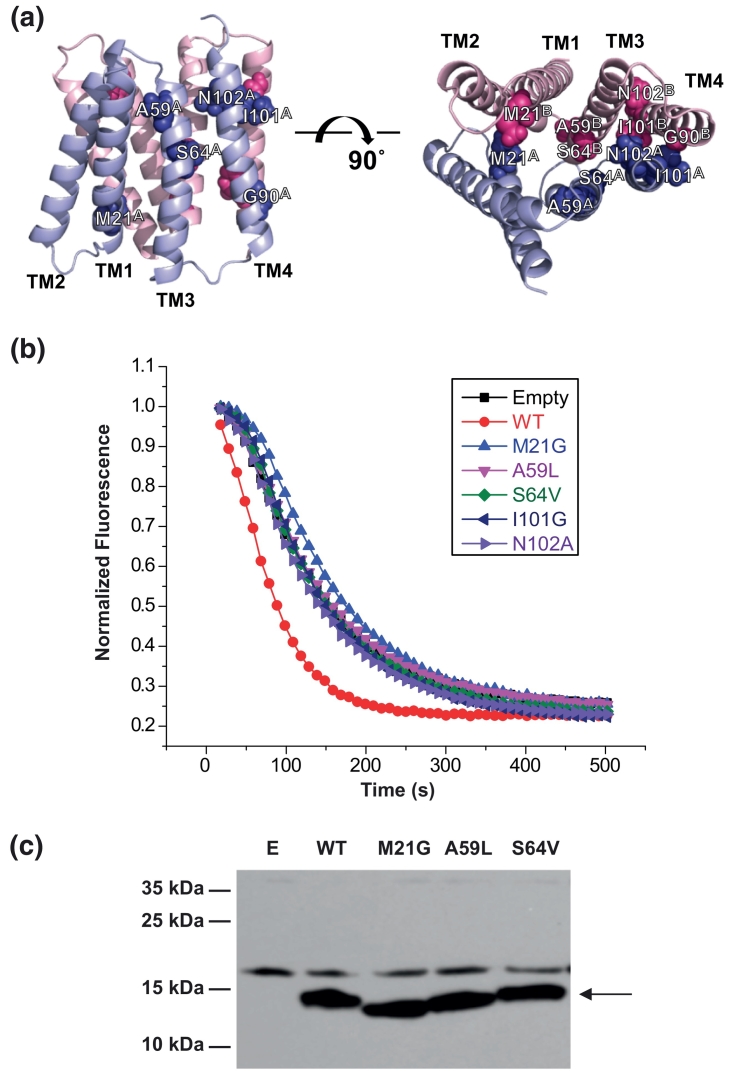
In-cell transport data confirm impaired transport phenotype. (a) Position of putative dynamics mutants on the structure, with wild-type residue identities indicated. (b) Efflux of ethidium from BL21 (DE3) *E. coli* cells transformed with either empty vector or the indicated EmrE mutant results in a decrease in fluorescence. (c) Western blot of selected EmrE mutants demonstrates that expression levels are similar to WT-EmrE.

Impaired drug-efflux activity could result from disruption of any one of the steps in the transport cycle. Alternating access between outward-open and inward-open states is essential for EmrE to confer drug resistance [6] but is not necessary for drug binding. Therefore, in order to identify mutants of EmrE more likely to have impaired alternating access rates, we first identified mutants with impaired resistance phenotypes we excluded mutations to residues known to be involved in drug binding and focused on sites that were not thought to be involved in direct interactions with substrates. The mutants chosen were M21G, A59L, S64V, G67A, G90V, G97V, I101G, N102A, and N102V. We then tested the ^3^H-TPP^+^-binding affinity of the selected mutants (Table 1). G67A and G97V were excluded at this stage because expression was very low, and they did not give good saturation binding curves, suggesting that they would not be able to be purified for structure determination. Four mutants showed affinities of ^3^H-TPP similar to wild type EmrE (S64V, I101G, N102A, N102V), two mutants had affinities 2- to 3-fold lower (M21G, G90V) and one was 10-fold lower (A59L). The location of all seven mutations is shown on the recently published refined atomic model of the complete structure of EmrE (Fig. 4a). M21G and A59L are located near the ends of the first and second TM helices, respectively. S64V is close to the ^65^GVG^67^ TM3 kink region, which is important for inward-open/outward-open interconversion and multidrug recognition according to cryo-EM and NMR studies [4], [5], [30], [32], [33]. G90V is located in the established G90/G97 TM4 dimerization motif [10], [13]. Finally, I101G, N102A, and N102V are located in TM4 but have not been directly implicated in dimerization.

**Table 1:** ^3^H-TPP^+^ binding to EmrE mutants in DDM^a^

EmrE mutant	*K*_d_ (nM)
Wild type	0.8 ± 0.1
M21G	4.0 ± 0.7
A59L	23.4 ± 2.4
S64V	1.0 ± 0.6
G90V	5.3 ± 0.6
I101G	2.6 ± 1.3
N102A	2.3 ± 0.4
N102V	1.2 ± 0.2

a Assays performed on ice, at pH 8. Binding affinity of TPP^+^ is in nM range due to higher pH and lower temperature [5], [31].

### Mutants with altered dynamics inhibit EmrE-mediated drug efflux

To confirm the impaired transport phenotype, the M21G, A59L, S64V, I101G, and N102A mutations were further characterized using an ethidium efflux assay in *E. coli* (Fig. 4b). This assay provided a kinetic assessment of EmrE activity, compared to the plate assay that simply reflected whether drug efflux was sufficient to enable survival over 24 h. The results confirmed that efflux of ethidium from *E. coli* was indeed slower for each of the EmrE mutants than for wild-type EmrE, and the slower efflux is not a result of decreased EmrE expression (Fig. 4c). This is consistent with the hypothesis that the impaired drug resistance activity of these EmrE mutants is due to a reduced rate of alternating access.

**Fig. 5:**
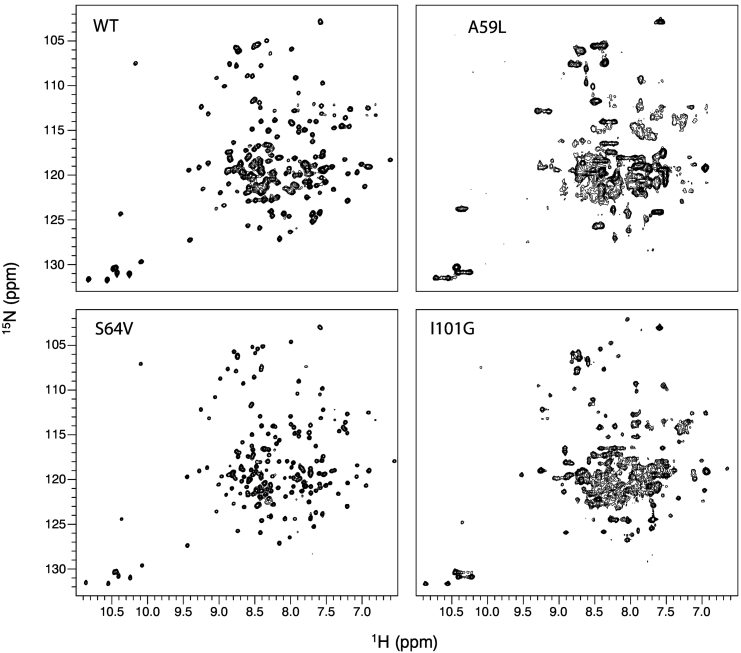
TROSY-HSQC spectra for TPP^+^-bound WT-EmrE and selected mutants. The S64V spectrum has sharper peaks than the WT spectrum, suggesting that this mutation decreases the dynamics of EmrE. A59L and I101G both exhibit significant line broadening, indicative of increased dynamics. Interestingly, peak doubling is still evident in the I101G spectrum, indicating that this TM4 mutation alters EmrE's dynamics without disturbing dimerization. The spectrum of all seven mutants can be found in Fig. S4.

The effect of the mutations upon EmrE dynamics was then assessed using NMR spectroscopy, a technique that is uniquely suited to provide information simultaneously about both protein structure and dynamics. We acquired ^1^H–^15^N TROSY HSQC spectra of each mutant in *q* = 0.33 DMPC/DHPC bicelles bound to tetraphenylphosphonium (TPP^+^) and compared them to TPP^+^-bound WT EmrE under the same conditions (Figs. 5 and S4). In the WT spectrum, two peaks are visible for each residue, reflecting the distinct chemical environments for each protomer in the asymmetric dimer. Peak doubling is still visible in the spectra for at least some residues in six mutants, indicating that the asymmetric EmrE dimer remains intact. The only exception is G90V-EmrE, which has fewer peaks and a poorly resolved spectrum, suggesting a loss of its unique tertiary structure of the asymmetric dimer. This is consistent with previous demonstrations that the dimer is destabilized when G90 or G97 is mutated [10], [13].

The ^1^H–^15^N TROSY spectra also provide substantial insight into protein dynamics. While each protomer in the asymmetric dimer of EmrE is in a unique chemical environment, the protomers swap conformations during alternating access [5]. The presence of pairs of relatively sharp peaks for each amino acid in the WT EmrE spectrum indicates that this alternating access rate is slow on the NMR timescale (*k*_ex_ < Δ*ω*, where Δ*ω* is the chemical shift difference between the two exchanging states) when TPP^+^ is bound. If this process were fast on the NMR timescale (*k*_ex_ > Δ*ω*), then the spectrum would reflect an average of the two chemical environments and only a single peak per amino acid would be visible. Dynamics on intermediate timescales result in significant broadening of the peaks in the NMR spectrum. Despite our expectations that impaired transport would arise from reduced dynamics, five mutants displayed *enhanced* dynamics. The ^1^H–^15^N TROSY spectra of I101G, A59L, N102A, and N102V (Figs. 5 and S4) displayed progressively increased line broadening, and each pair of peaks shifted toward each other, eventually coalescing into a broad single peak for many residues. It is important to note that the effect of dynamics on peak widths and intensities is not limited to alternating access conformational exchange. The enhanced line broadening may also reflect greater structural heterogeneity or increased motion within the “ground-state” structure. This is a more likely explanation for M21G, which has significant loss of peak intensity while still having well-resolved peak doubling.

### Importance of the TM3 kink for alternating access and drug transport by EmrE

**Fig. 6:**
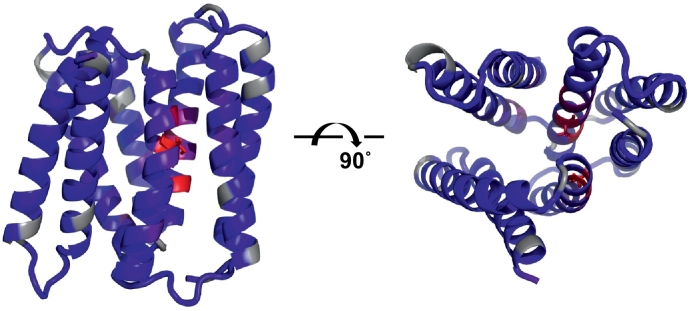
S64V mutation perturbs TM3 kink region locally. Chemical shift differences between WT and S64V-EmrE were plotted onto structure. The largest perturbations are seen in the immediate vicinity of the TM3 kink. Nevertheless, several smaller changes are seen throughout the protein, suggesting several long-range allosteric effects of the S64V mutation.

In contrast to all other mutants, S64V-EmrE displayed pairs of sharp, well-resolved peaks throughout the spectrum. The ^1^H–^15^N TROSY spectrum of this mutant was similar to WT EmrE, indicating that it has a very similar asymmetric dimer structure. Indeed, chemical shift changes were greatest in the immediate vicinity of the mutation, as shown on the three-dimensional structure in Fig. 6. Since the chemical shifts reflect the unique environment of each nucleus, these data indicated that the three-dimensional structure of the asymmetric EmrE homodimer was very similar in WT EmrE and S64V-EmrE.

**Fig. 7:**
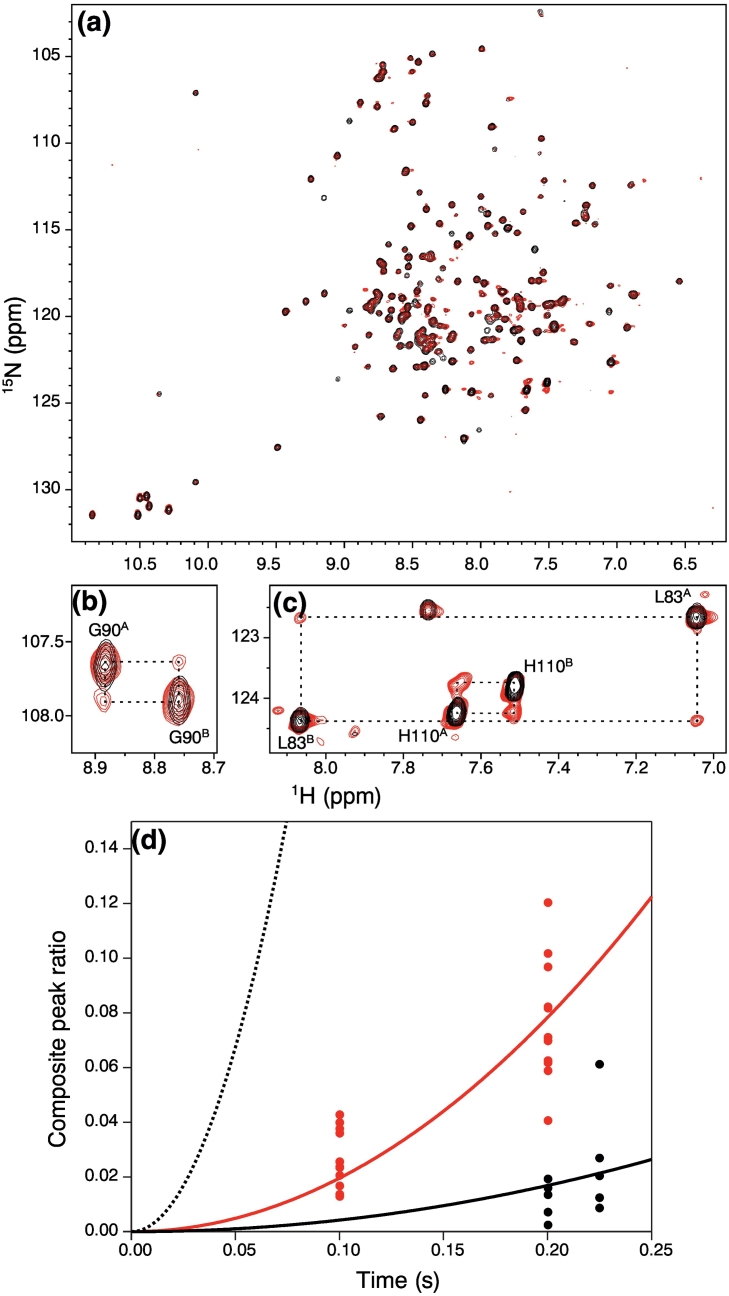
S64V mutation slows down conformational interconversion rate of EmrE. (a) TROSY-HSQC (black) overlay with a ZZ-exchange plane with a mixing time of 200 ms (red) for TPP^+^-bound S64V-EmrE. (b) Detail showing the pair of G90 peaks and their cross-peaks in the ZZ-exchange spectrum. (c) Detail showing the pairs of L83 and H110 peaks and their cross-peaks in the ZZ-exchange spectrum. (d) Composite peak ratio fitting as a function of mixing time for TPP^+^-bound S64V-EmrE in DMPC (solid black) or DLPC (solid red) bicelles. The dotted line indicates the fit of TPP^+^-bound WT EmrE (data in Fig. S5).

To determine whether S64V exhibited slower dynamics, NMR exchange spectroscopy was used to directly determine the alternating access rate. For a static structure, the ZZ-exchange spectrum will be identical to the ^1^H–^15^N TROSY spectrum. However, if alternating access occurs during the delay period in the ZZ-exchange experiment, additional cross-peaks appear in the spectrum (Fig. 7a–c). By varying the mixing time, the build-up of cross-peaks and decay of autopeaks can be fit to quantitatively determine the kinetics of alternating access (Fig. 7d), as we have previously shown for WT EmrE [5]. The S64V alternating access rate measured with these experiments is 0.6 ± 0.1 s^−1^, 8-fold slower than WT EmrE under the same conditions (4.7 ± 0.6 s^−1^) [33]. In addition, the individual peaks in the spectra are even more intense and uniform in shape, suggesting less structural plasticity in this mutant on all timescales.

Finally, we used isothermal titration calorimetry (ITC) data to determine the affinity of S64V for several different drug substrates. The data show that S64V-EmrE binds TPP^+^, methyltriphenylphosphonium (MeTPP^+^), and ethyltriphenylphosphonium (EtTPP^+^) with affinities nearly identical to WT EmrE (Tables 2, S1, and S2; and Fig. S6). This is consistent with the NMR chemical shift data indicating very little difference in the structure of this mutant and confirms that impaired transport is not due to difference in drug binding affinity. Thus, we have successfully discovered a dynamics mutant that has a reduced rate of alternating access without disrupting the structure or substrate affinity of EmrE.

**Table 2:** S64V- and WT-EmrE drug binding affinity by ITC^a^

Substrate	*K*_d_ (μM)	Δ*H* (kJ/mol)	n
S64V (DMPC/DHPC bicelles)			
TPP^+^	0.6 ± 0.1	− 52 ± 2	0.50 ± 0.03
EtTPP^+^	11.4 ± 0.1	− 51 ± 5	0.51 ± 0.04
MeTPP^+^	60 ± 10	− 40 ± 10	0.49 ± 0.01
WT (DMPC/DHPC bicelles)			
TPP^+^	0.5 ± 0.1	− 29 ± 4	0.49 ± 0.03
WT (DLPC/DHPC bicelles) [33]			
TPP^+^	0.45 ± 0.01	− 22 ± 1	0.49 ± 0.05
EtTPP^+^	21.8 ± 0.7	− 16.6 ± 0.2	0.57 ± 0.04
MeTPP^+^	130 ± 20	− 12.7 ± 0.2	0.60 ± 0.04

a Assays performed at 45 °C, pH 7.

## Discussion

In this study, we aimed to identify mutations that suppressed the ability of EmrE to exchange between conformations open to either side of the membrane. To this end, we performed the single most extensive mutagenesis scan of EmrE to date, testing the effect of mutations to one of three amino acids (alanine, glycine, or valine) at each position on resistance to three canonical EmrE substrates (ethidium, acriflavine, or methyl viologen). From this screen, we selected several mutants that exhibited impaired drug resistance but normal drug binding for further dynamics study. In a kinetic assay, all of the mutants had reduced rates of proton motive force-driven drug efflux in *E. coli*. Alternating access in the drug-bound state is the rate-limiting step in the transport cycle of TPP^+^ by wild-type EmrE [21], [34]. Thus, we hypothesized that the reduced rate of drug efflux by these mutants was due to a decrease in the rate of this critical step. Surprisingly, the rate of net drug efflux from *E. coli* for our mutants does not correlate simply with the rate of alternating access observed by NMR.

How might the rate of drug efflux be reduced despite an increased rate of alternating access? EmrE's transport mechanism is complex [21], [35], [36], so it is possible that the mutations drastically reduced the rates of steps other than alternating access, thereby changing which step is rate limiting. However, in light of the overall increased dynamics displayed by the mutant spectra, it is important to note that decreased transporter dynamics are not required for a decreased rate of transport. Efficient drug efflux requires allosteric communication between various regions of the protein, such that large-scale dynamic motions, including the loop and helix rearrangements necessary for alternating access, are properly coordinated to binding of drug and proton. Loss of this coordination could increase the rate of drug or proton leak pathways, reducing the efficiency of transport even when alternating access is fast [31].

Unfortunately, the lack of a high-resolution EmrE structure precludes a deep understanding of its allosteric couplings. Nevertheless, there are several examples of known allosteric mechanisms that are essential for proper transport. The first and most important is the relationship between the alternating access rate of EmrE and the occupancy of the E14 binding site. This rate varies over several orders of magnitude depending on the identity of bound drug, while in the absence of drug, it increases upon protonation [22], [33], [37]. A second allosteric coupling involves the TM2–3 loop, which exhibits increased dynamics upon protonation of E14. This loop is proposed to be involved in a latching mechanism, wrapping around TM1 to stabilize the closure of the transport pore on one side of the membrane and prevent proton leak. Mutation of this loop to destabilize the latch can abolish drug resistance (I54G, repeated here in Table S1) and increase the alternating access rate (I54L) [37]. A final set of couplings involve the TM1–2 and TM3–4 loops. In an electron paramagnetic resonance study, it was shown that these loops close to form an occluded state upon protonation of a residue apart from E14 [36], which our NMR data suggest is H110 [22], [35]. Recently, we demonstrated further coupling between the C-terminal tail, which includes H110, and the occupancy of the E14 binding site. Consideration of these couplings led to our proposal of an allosteric gating mechanism to prevent futile proton transport in the absence of drug [35].

It seems likely that mutations that increase the alternating access rate of EmrE disrupt one or more of these mechanisms, resulting in the impaired drug resistance phenotype. A59L is located at the beginning of TM3, possibly disrupting stable closure of the TM2–3 loop. Similarly, M21G is located at the end of TM1 and could disturb EmrE gating by disrupting the coupling of TM1, which contains the critical E14 residue, with the rest of the transporter. Previous comparison of the NMR chemical shifts of TPP^+^-bound EmrE showed very little difference between TM1 and TM2 in the two protomers of the asymmetric homodimer [32], suggesting that the first few helices move together as the protein switches between open-in and open-out conformations. Loss of this coordinated movement could disrupt the transition and gating of EmrE.

It is more difficult to rationalize the effects of I101G, N102A, and N102V within our current understanding of EmrE structure and function. The primary role of TM4 is thought to be dimerization [10], [38], holding the small EmrE homodimer together, while it undergoes the large-scale conformational changes needed to alternate access. However, mutations at positions 101 and 102 did not disrupt the EmrE dimer in previous mutagenesis studies and do not disrupt dimerization in the mutants examined here [13].

The answer could lie in the importance of the ^65^GVG^67^ motif, which forms a hinge point in TM3 [10]. In the available moderate-resolution structures, this kink is only found in monomer A of the EmrE homodimer [3], [4], [18]. The NMR chemical shift data confirm this difference in TM3 structure between the two halves of the dimer [5]. Thus, the TM3 helices must alternately kink and straighten as EmrE transitions between open-in and open-out conformations during each transport cycle. It is perhaps not surprising then that the one slow dynamics mutant of EmrE we identified, S64V, is adjacent to this motif. Interestingly, residues 101 and 102 also pack against this region. Re-examining our previous studies of WT EmrE bound to diverse substrates confirms the coupling between TM3 and this segment of TM4: large chemical shift changes are observed for TM4 residues at the level of the TM3 kink when EmrE is bound to different substrates [33]. We previously attributed this to indirect effects from structural changes in the TM3 kink that are necessary for EmrE to accommodate diverse substrates. However, the dramatic phenotypes of I101G, N102A, and N102V presented here suggest that mutation of these residues may instead disrupt a critical function of the TM3 kink. We are currently exploring the effects of additional substitutions at position 64 and the effect of the S64V mutation on each step in the transport cycle. Ultimately, a deep understanding of the importance of this region, and of EmrE's broader transport mechanism, will require a high-resolution structure. This effort is underway, facilitated by the slow dynamics of the S64V mutant presented here.

## Materials and Methods

### Screening for putative dynamics mutants

Every codon in the gene encoding EmrE was mutated using degenerate oligonucleotides that converted a given codon to GBN, where B either is T, C, or G and N is any base. Thus, every amino acid residue was changed either to Val, Ala, or Gly [29]. The mutagenic reactions (QuikChange II; Agilent) were performed on the wild-type *EmrE* gene inserted under the control of the *tac* promoter in plasmid pKK223–3 (making plasmid pKK56 [1]), transformed into *E. coli* strain JM109 and colonies grown on 2xTY agar plates containing 100 μg/ml ampicillin. Eight colonies were picked from each mutagenic reaction, grown overnight at 37 °C in 1 ml 2xTY in a deep-well block, and then this culture was diluted 1000-fold. The ability of each EmrE mutant to transport substrates out of *E. coli* was then assessed by spotting 5 μl of the diluted culture onto 2xTY agar plates either containing methyl viologen (50 or 200 μM), acriflavine (116 or 463 μM), ethidium bromide (400 or 1500 μM), or control plates containing no substrate. DNA sequencing was performed in parallel to determine which mutations were present in each EmrE mutant. In the first round of mutagenesis, 109 amino acid residues corresponding to residues 2–110 of EmrE were mutated, and 276 of the possible 327 mutants were obtained (Figs. S1 and S2). The remaining 51 mutants were made using oligonucleotides designed specifically to introduce a single amino acid residue, sequenced and then tested for their growth phenotype on the three substrates (Fig. S3). Five mutants were not made (F44A, W63 V, F79G, D84A, I88G). Growth was scored as robust at both concentrations of a given substrate (++), growth at both substrate concentrations but notably reduced relative to WT at the higher concentration (+), growth only at the lower substrate concentration (+/−), or no growth at either substrate concentration (−) for each of the three different substrates. Neither JM109 nor JM109(pKK56) grew in the presence of any of the substrate concentrations tested. Selected mutants with impaired transport, as indicated by severe defects in the growth assays, were then checked for substrate binding using [^3^H]-TPP^+^ binding assays performed with the selected EmrE mutants purified in DDM detergent using previously published methods [39]. Data were gathered from a single experiment performed in triplicate. Reported errors are standard error of mean.

### ConSurf analysis

Sequence conservation analysis using ConSurf [40], [41], [42] was performed on the EmrE amino acid sequence using one iteration of HMMER to search for homologs with *E*-value cutoff of 0.0001 against protein database of UNIREF-90. A total of 300 sequences with that sample the list of homologs to reference sequence were selected out of homology search. These sequences were aligned using MAFFT. Calculated Bayesian conservation scores were mapped on the structure.

### Sample preparation

WT and mutant EmrE was expressed, purified, and reconstituted into DLPC or DMPC liposomes as previously described [5], [33]. Detergent was removed by Amberlite® XAD®-2 resin, and 6:0 DHPC was added to form isotropic bicelles as described in Ref. [43]. Single point mutants were constructed using QuikChange (Stratagene).

### In-cell assay

These assays were carried out using BL21(DE3) *E. coli* transformed with empty pET15b vector or pET15b-EmrE with the specified WT or mutant sequence. The cells were grown in M9 minimal media with 100 μg/ml of ampicillin at 37 °C until the OD_600_ reached 0.4. Then cells were induced with 0.33 mM IPTG at the same temperature for 30 min. After induction, cell density was adjusted to OD_600_ = 0.4 followed by incubation with 2.5 μM ethidium bromide and 40 μM carbonyl cyanide p-chlorophenylhydrazone for an hour to load ethidium bromide into the cells. The cell cultures were then stored on ice until assays were complete. For each experiment, 2 ml of cell culture was spun down and immediately resuspended in 1 ml fresh M9 media with 2.5 μM ethidium bromide. Fluorescence of ethidium bromide was monitored with an excitation wavelength at 545 nm and emission wavelength at 610 nm. The time course of fluorescence was plotted after normalization to the initial value of each run.

### Western blot analysis of EmrE expression levels

Cells prepared for the in-cell transport assay as described above were induced and adjusted to OD_600nm_ = 0.4. A 1-ml aliquot of this cell suspension was pelleted by centrifugation and resuspended in 40 μl of SDS-PAGE sample buffer. A 5-μl sample was loaded on a 4%–12% Bis–Tris NuPAGE gel (Thermo Fisher) and, following electrophoresis, transferred onto Immobilon PVDF membrane (EMD-Millipore). The blot was probed with anti-His_6_-HRP conjugated antibody (QIAGEN) at 1:10,000 dilution, following the manufacturer's instructions. Bands were visualized with ECL Prime Western blotting kit (GE) according to the standard protocol. HyBlot Films (Denville) were exposed for 3 min and captured with EZ doc imaging system (BioRad).

### NMR spectroscopy and data analysis

NMR data were collected using samples with 0.8–1.5 mM ^2^H,^15^N EmrE in DMPC/DHPC bicelles (*q* = 0.33, with a protein to DMPC molar ratio of 1:50) and 100 mM Mops, 10–30 mM NaCl, 2 mM TCEP, and 8%–10% D_2_O (pH 7) at 45 °C on a Varian 700-MHz spectrometer with a room temperature probe unless otherwise noted. All NMR spectra were processed with NMRPipe [44] and analyzed in CcpNmr Analysis [45]. For the TPP^+^-bound EmrE, 2 mM TPP^+^ was added to ensure EmrE saturation. 2D ^1^H,^15^N TROSY-HSQC and TROSY-selected ZZ-exchange experiments [46] with a lipid flip-back pulse [5] were carried out with a recycle delay of 2 s and 128–144 increments.

Chemical shift differences (*Δδ*) between TPP^+^-bound WT and S64V EmrE were calculated as a weighted average of the differences in amide proton (*Δδ*_H_) and nitrogen (Δδ_N_) chemical shifts according to:

(1) $$∆\delta =\sqrt{{{∆\delta }_{H}}^{2}+{\left(0.154\;{∆\delta }_{N}\right)}^{2}}$$

The conformational interconversion rate, *k*_conf_, was analyzed from the ZZ-exchange data as previously described [47] using the composite peak ratio method with an 11.1-ms offset time, *t*_0_, to account for the back-transfer time in the pulse sequence [22]. The composite peak ratios of intensities of the auto-peaks (*I*_AA_, *I*_BB_) and cross-peaks (*I*_AB_, *I*_BA_) were fit to the following equation as a function of the delay time, *t*:

(2) $$\Xi \left(t\right)=\frac{I_{\mathrm{AB}}\left(t\right)I_{\mathrm{BA}}\left(t\right)}{I_{\mathrm{AA}}\left(t\right)I_{\mathrm{BB}}\left(t\right)-I_{\mathrm{AB}}\left(t\right)I_{\mathrm{BA}}\left(t\right)}≅k_{\text{conf}}^{2}{\left(t-t_{0}\right)}^{2}$$

For TPP^+^-bound EmrE, at least two planes were collected with different mixing times and the mixing times were adjusted according to the conformational interconversion rate of each mutant. For S64V, the slow rate of alternating access requires long mixing times in order for measurable cross-peak build up and the large size of bicelle-solubilized EmrE leads to relatively fast relaxation, which limits the total possible mixing time. Thus, for TPP^+^-bound S64V-EmrE, mixing times of 100 and 200 ms were used in DLPC bicelles and 200 and 225 ms in DMPC bicelles. For TPP^+^-bound WT-EmrE in DMPC bicelles, conformational exchange is faster, and four planes with mixing times of 20, 50, 90, and 125 ms were used to quantify the rate of alternating access. The rate was determined by global fitting of all residues with resolved auto- and cross-peaks in the spectra, and the standard deviation of the rates determined by separately fitting individual residues was used to estimate the error of the rates.

### ITC

All ITC experiments were performed on a TA instruments Nano-ITC calorimeter as previously described [33]. Data were fit to a model of ligand binding to *n* independent and identical sites plus a constant baseline due to mixing using the NanoAnalyze software EmrE was reconstituted into DMPC/DHPC (*q* = 0.33) isotropic bicelles in 20 mM potassium phosphate and 20 mM NaCl (pH 7) at 45 °C and loaded into the cell at 40–60 μM for TPP) titrations and 500 μM for titration with MeTPP^+^, or EtTPP^+^. TPP^+^ concentration in the syringe was 150–200 μM. MeTPP^+^ and EtTPP^+^ concentrations were 2–4 mM with buffer and bicelle conditions that exactly matched the protein solution in the cell. Titrations replicates are listed in Tables S1 and S2. Reported errors are standard error of mean.

### NMR assignment deposition

NMR assignments were deposited to the Biological Magnetic Resonance Database under accession number 27902.

The following are the supplementary data related to this article.

Supplementary figures and raw data Image 1

Supplementary Data Table 1 Scoring of mutant EmrE resistance phenotypes. Supplementary Data Table 1
