# Supplementary material for: Identification of an Alternating-Access Dynamics Mutant of EmrE with Impaired Transport
Source: J Mol Biol. 2019 Jul 12;431(15):2777–89. doi: 10.1016/j.jmb.2019.05.035 (PMC6599891; doi:10.1016/j.jmb.2019.05.035)
Supplement: Supplementary file 1 — Supplementary figures and raw data [file mmc1.docx]

**Figure S1 Resistance screen of substitutions at positions 2-56** Mutations were made with degenerate primers, and sequencing was performed in parallel with resistance screen

**Figure S2 Resistance screen of substitutions at positions 57-110** Mutations were made with degenerate primers, and sequencing was performed in parallel with resistance screen

**Figure S3 Resistance screen of remaining substitutions** Mutations were made using QuikChange


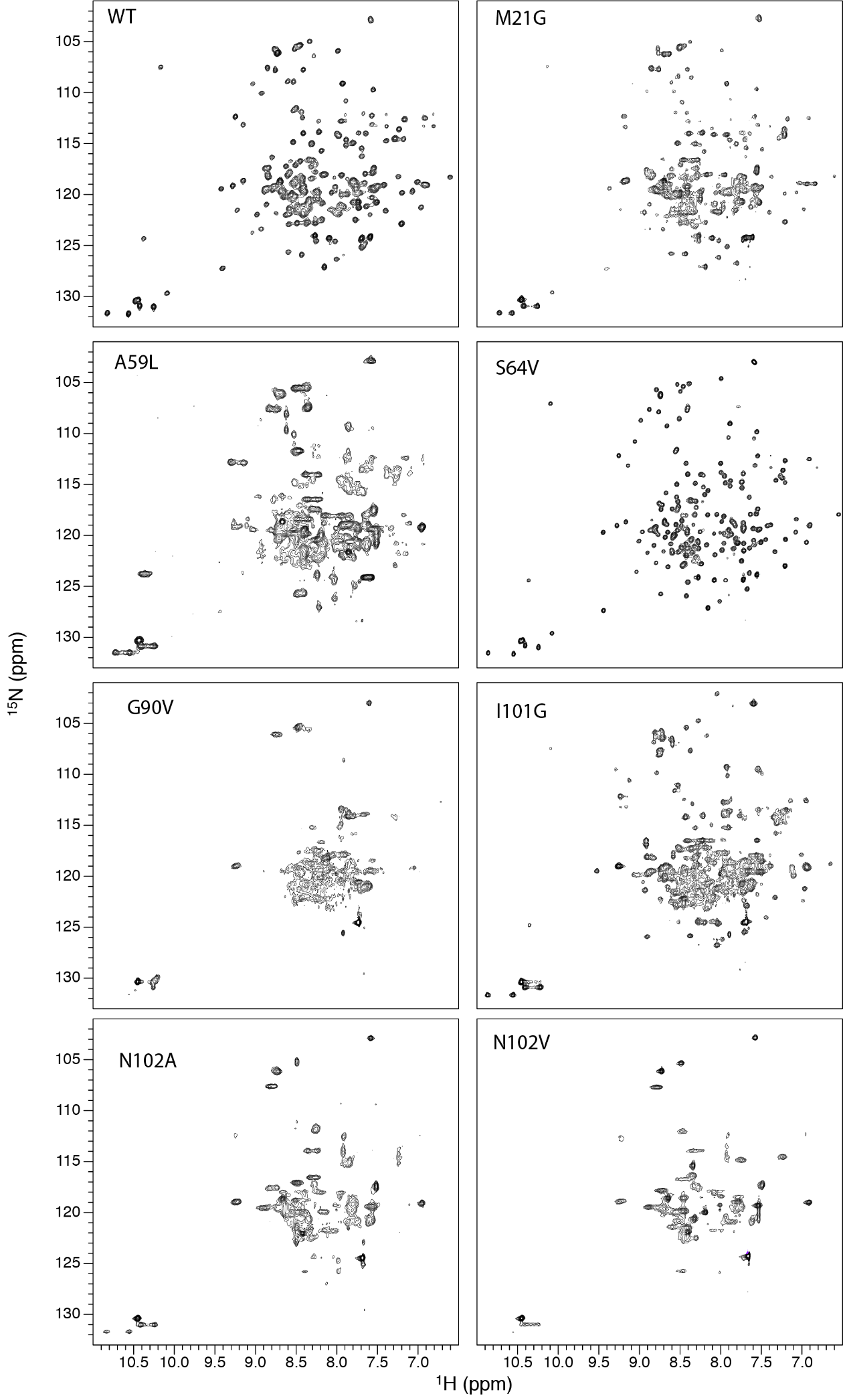


**Figure S4 TROSY-HSQC spectra for TPP^+^-bound WT-EmrE and mutants**

**
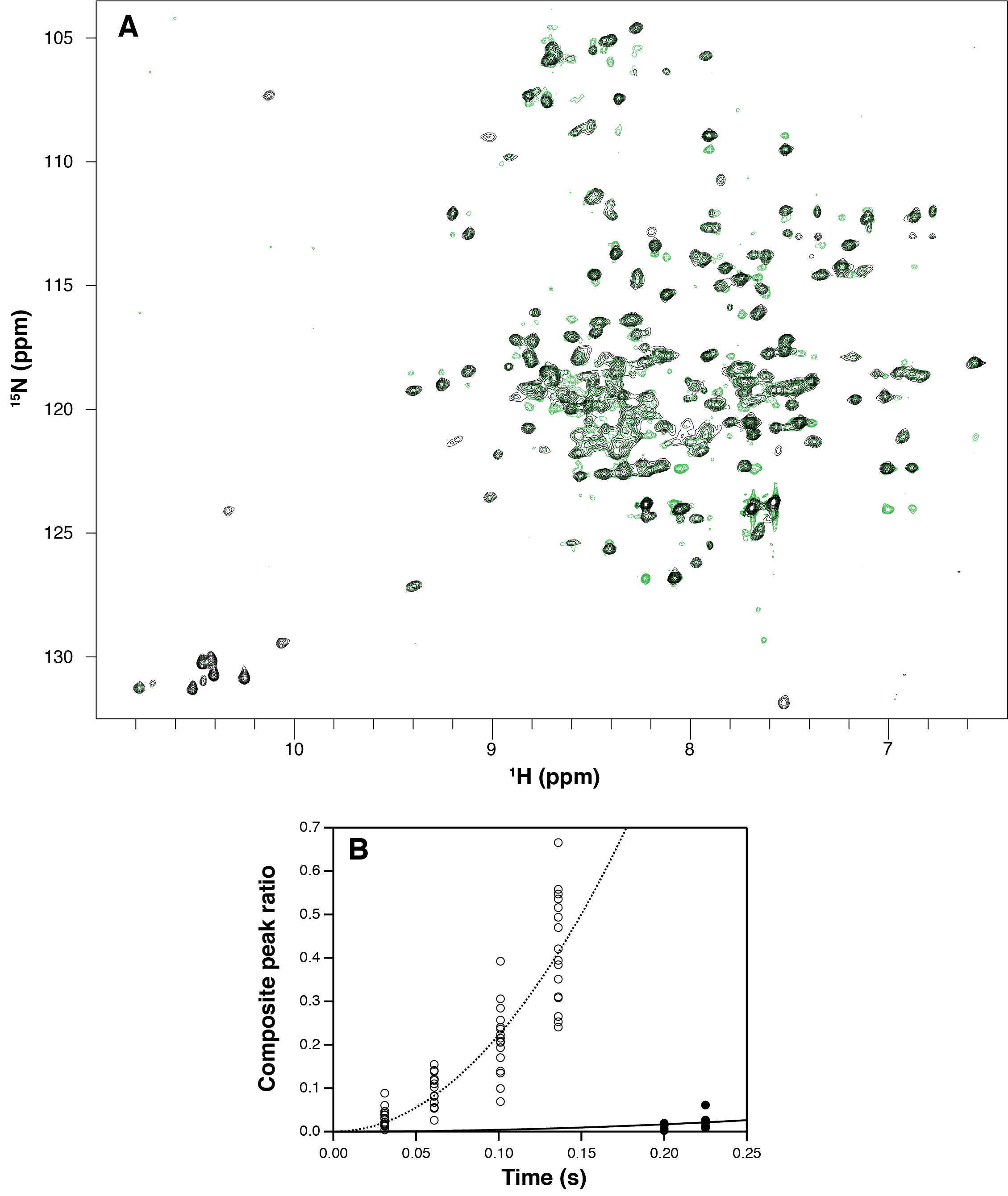
**

**Figure S5. Alternating access rate of TPP^+^-bound WT and S64V-EmrE in DMPC/DHPC isotropic bicelles.** *A*, TROSY-HSQC (black) overlay with a ZZ-exchange plane with a mixing time of 90 ms (green) for TPP^+^-bound WT EmrE in DMPC bicelles. *B*, composite peak ratio fitting as a function of mixing time for TPP^+^-bound WT EmrE in DMPC bicelles (open circles, dotted line) and S64V-EmrE in DMPC bicelles (filled circles, solid line). The data is shown for multiple residues from across the protein, and the fitted line is a global fit.


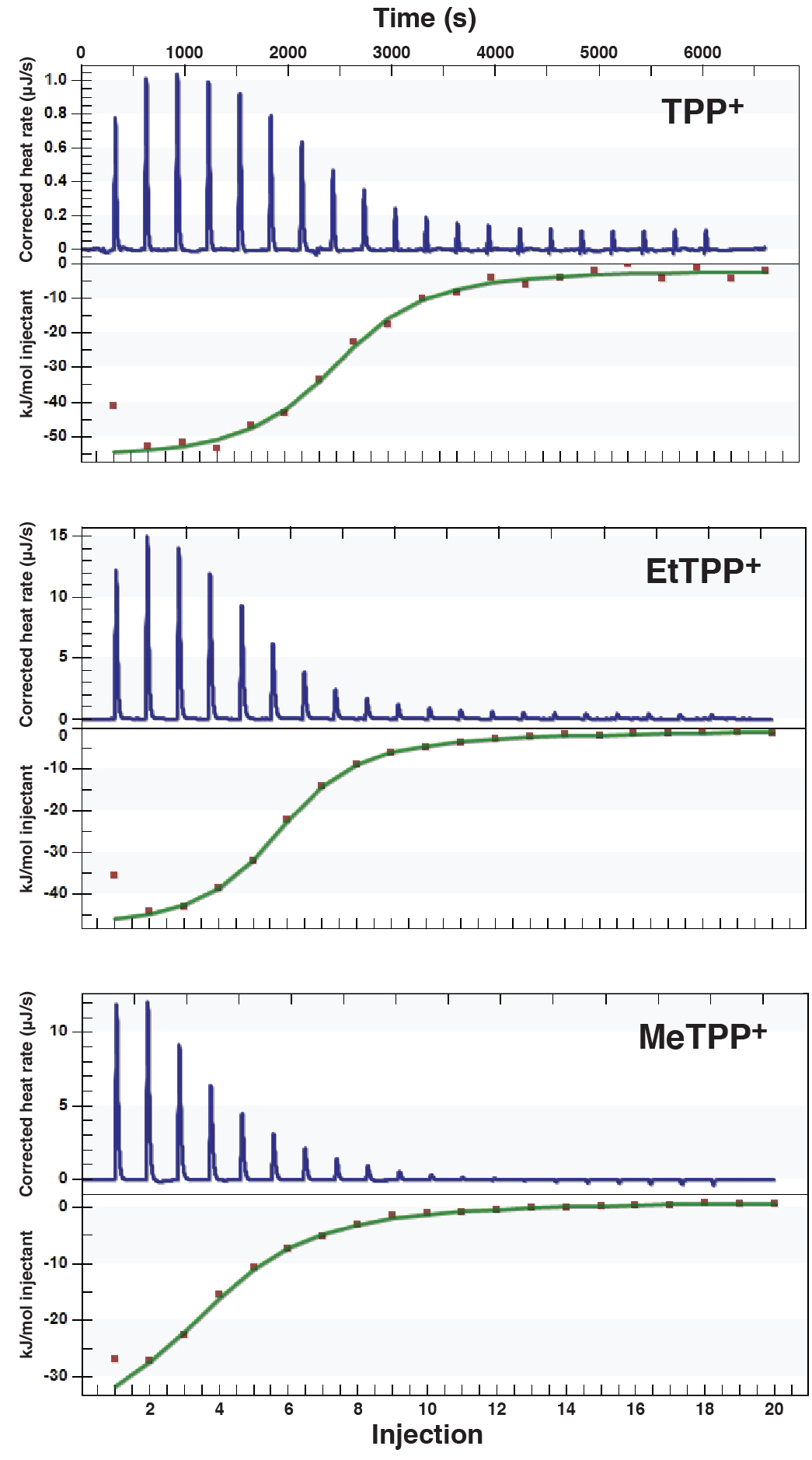


**Figure S6** *S64V-EmrE binds a variety of ligands* Representative traces of isothermal titration calorimetry experiments for S64V binding to tetraphenylphosphonium (TPP^+^), methyltriphenyl-phosphonium (MeTPP^+^), and ethyltriphenyl-phosphonium (EtTPP^+^).

**Table S1. ITC Data for TPP^+^ Binding to EmrE in Isotropic Bicelles***

|  | **K_d_ (µM)** | **∆H (kJ/mol)** | **n** |
| --- | --- | --- | --- |
| **S64V-EmrE** | 0.628 | -56.0 | 0.465 |
|  | 0.483 | -51.6 | 0.535 |
|  | 0.500 | -50.9 | 0.526 |
|  | 0.680 | -50.9 | 0.503 |
|  | 0.686 | -53.0 | 0.469 |
| **WT-EmrE** | 0.589 | -34.3 | 0.455 |
|  | 0.325 | -28.7 | 0.506 |
|  | 0.395 | -27.8 | 0.477 |
|  | 0.569 | -23.6 | 0.531 |

*q=0.33 DMPC/DHPC isotropic bicelles at pH 7

**Table S2. ITC Data for Different Ligands Binding S64V-EmrE***

|  | **K_d_ (µM)** | **∆H (kJ/mol)** | **n** |
| --- | --- | --- | --- |
| **EtTPP^+^** | 12.9 | -46.8 | 0.474 |
|  | 20.5 | -62.7 | 0.568 |
| **MeTPP^+^** | 56.2 | -41.9 | 0.474 |
|  | 46.3 | -34.5 | 0.481 |

*q=0.33 DMPC/DHPC isotropic bicelles at pH 7
